# High performance supercapacitors driven by the synergy of a redox-active electrolyte and core–nanoshell zeolitic imidazolate frameworks†

**DOI:** 10.1039/d4na00805g

**Published:** 2025-02-10

**Authors:** Mansi, Vishal Shrivastav, Prashant Dubey, Aristides Bakandritsos, Shashank Sundriyal, Umesh K. Tiwari, Akash Deep

**Affiliations:** a CSIR-Central Scientific Instruments Organisation (CSIR-CSIO) Chandigarh 160030 India umeshtiwari@csio.res.in; b Academy of Scientific and Innovative Research Ghaziabad 201002 India; c Regional Center of Advanced Technologies and Materials, The Czech Advanced Technology and Research Institute (CATRIN), Palacký University Olomouc Šlechtitelů 27 779 00 Olomouc Czech Republic shashank.sundriyal@upol.cz; d Advanced Carbon Products and Metrology Department, CSIR-National Physical Laboratory (CSIR-NPL) New Delhi 110012 India; e Nanotechnology Centre, Centre for Energy and Environmental Technologies, VŠB – Technical University of Ostrava 17. listopadu 2172/15 708 00 Ostrava-Poruba Czech Republic; f Institute of Nano Science and Technology (INST) Sector-81 Mohali 140306 Punjab India akashdeep@inst.ac.in

## Abstract

The selection of appropriate electrolytes plays a crucial role in improving the electrochemical performance of the supercapacitor electrode. The electrolyte helps to select an appropriate potential window of the device, which is directly related to its energy density. Also, the selection of an appropriate electrode material targets the specific capacitance. Therefore, in this work, we targeted an electrode material based on a ZIF-8@ZIF-67 (Z867) core–nanoshell structure and tested its performance in redox active electrolyte (RAE), *i.e.*, 0.2 M K_3_[Fe(CN)_6_] in 1 M Na_2_SO_4_. The synergy between the core–nanoshell electrode having ZIF-8 as a core and ZIF-67 as a nanoshell along with RAE further complements the redox active sites, resulting in the improved charge transport. Therefore, when the Z867 core–nanoshell electrode is tested in a three-electrode system, it outperforms pristine ZIF-8 and ZIF-67 electrode materials. The working electrode modified with the Z867 core–nanoshell showed a maximum specific capacitance of 496.4 F g^−1^ at 4.5 A g^−1^ current density with the RAE, which is much higher than that of the aqueous electrolyte. A Z867-modified working electrode was assembled as the positive and negative electrode in a symmetrical cell configuration to create a redox supercapacitor device for practical application. The constructed device displayed maximal energy and power densities of 49.6 W h kg^−1^ and 3.2 kW kg^−1^ respectively, along with a capacitance retention of 92% after 10 000 charge–discharge cycles. Hence, these studies confirm that using RAE can improve the electrochemical performance of electrodes to a greater extent than that of aqueous electrolyte-based supercapacitors.

## Abstract 2

A Z867 core–shell MOF electrode symmetrical device delivered superior electrochemical performance (49.6 W h kg^−1^ at 3.2 kW kg^−1^) in a redox-active electrolyte (RAE).

## Graphical abstract

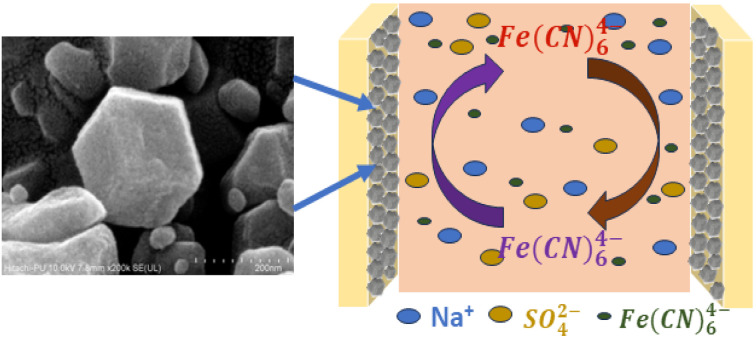

## 1. Introduction

The efficient storage of energy has been a serious concern in the modern world as the demand for power supply has increased due to the growing need for high-tech electric vehicles, mobile phones, portable electronic devices, *etc.* Such devices necessitate high power supply systems with high energy and power density. Nonetheless, emerging energy storage devices like fuel cells and lithium-ion batteries exhibit high energy density but still have issues with high power supply.^1–3^ In this regard, electrochemical supercapacitors have drawn a lot of attention compared to conventional capacitors because of their quick charge–discharge properties, high power density, and outstanding cycling stability. Supercapacitors have been used in various applications, including wearable electronics, paper-like electronics, flexible biomedical devices, military and space equipment, and hybrid electric vehicles. These applications benefit from supercapacitors' high-power capability, exceptional cycle life, and reliability.^4^ However, SCs' relative lack of energy density compared to conventional rechargeable batteries prevents their future advancement and practical use. Hence, a crucial aspect in developing advanced energy storage devices involves enhancing their energy density while preserving high-power density. According to the formula *E* = 0.5*CV*^2^, it is established that energy density is influenced by the capacitance of electrode materials and the potential window of a supercapacitor (SC). Increasing capacitance and elevating cell voltage effectively is a viable strategy for achieving heightened energy density. The specific capacitance of the electrode is increased by selecting an appropriate electrode material which should possess a porous structure with high specific surface areas, hierarchical pore size distribution, and enough electroactive sites.^5^ In this context, metal–organic frameworks (MOFs), an innovative category of porous materials showcasing versatile functionalities, have garnered significant interest in supercapacitor (SC) applications. This enthusiasm stems from their notable attributes, including a high specific surface area, controllable pore size and topologies, well-ordered structure, and open-metal sites.^6^ Using pristine MOFs or MOF-derived materials as SC electrodes (such as porous carbons and metal oxides) has been thoroughly reported.^7,8^ The progress of MOFs in energy applications has been spectacular during the past few years, yet low conductivity and arbitrary orientations are two of the key drawbacks that limit their potential usage in SC applications. Zeolitic imidazolate frameworks (ZIFs), a subfamily of MOFs, are composed of tetrahedral MN_4_ (M = Co, Zn, *etc.*) units connected by imidazolate ligands.^9,10^ Due to their distinct structural qualities, which include enhanced thermal and chemical stability in organic and aqueous fluids, ZIFs can be employed to improve the cycling performance of the supercapacitor under the assumption of retaining a higher specific capacitance.^11^ Additionally, the ZIF's metal ions' redox activity may provide an electron transport pathway and reduce charge carrier diffusion routes. Numerous research studies have shown the usage of ZIFs and materials made from ZIFs as potential electrodes in energy storage devices or other specific applications, taking into account the aforementioned criteria.^12,13^ In this context, Wang *et al.* reported a novel water-mediated transformation of ZIF-67 into Ni–Co layered double hydroxide (LDH) hollow microspheres.^14^ The resulting Ni–Co LDH hollow microspheres exhibit a unique hierarchical structure with high surface area and porosity. This architecture improves electrolyte penetration and ion diffusion, critical for enhancing electrochemical performance. The synergistic effect between nickel and cobalt in the LDH contributes to superior pseudocapacitance (1530 F g^−1^ at 1 A g^−1^) due to redox-active transitions. In another report, NiCo_2_O_4_ exhibits diverse hierarchical structures such as nanosheets, nanorods, and nanospheres, depending on reaction time and temperature. These structures provide high surface area, abundant active sites, and enhanced porosity, promoting better electrolyte accessibility and ion transport.^15^

However, exploitation of pristine ZIFs in energy storage applications has few limitations. They often exhibit poor electrical conductivity, limiting their performance in energy storage applications. The insulating nature of the organic ligands in ZIFs hinders efficient electron transport, leading to lower charge/discharge rates and overall energy storage capabilities.^16^ Also, during the charge/discharge cycles, pristine ZIFs may undergo significant volume expansion and contraction, leading to mechanical instability. This can result in the pulverization of the material, compromising its structural integrity and overall cycling stability.^17^ Pristine ZIFs might also have a limited specific surface area, which can impact their overall energy storage capacity. This limitation arises from the inherent structure of ZIFs and can restrict the accessibility of active sites for charge storage.^18^ ZIF-based core–nanoshell structures overcome these limitations with the synergy of exploitation of two different metal ions. Incorporating ZIFs into a core–nanoshell structure, particularly with conductive materials as the nanoshell, can significantly enhance the overall electrical conductivity. This improvement facilitates faster electron transport, leading to improved charge/discharge rates and overall energy storage performance.^19^ The core–nanoshell structure provides mechanical support to the ZIFs, mitigating the volume expansion issues observed in pristine ZIFs. The outer nanoshell acts as a buffer, preventing the pulverization of the ZIFs and ensuring structural integrity during repeated cycling.^20^ The core–nanoshell structure can provide a larger surface area and expose more active sites of ZIFs, leading to enhanced charge storage capacity. This increased accessibility improves the overall energy storage performance of the material.^21^ Further structural strategies for a core–nanoshell MOF as a novel energy storage electrode material involve hierarchical porosity enhancement, composition modulation, core–nanoshell architecture design, surface functionalization, and nano-structuring. Hierarchical porosity, achieved through controlled synthesis methods, enhances ion diffusion and electrolyte accessibility, improving charge storage capacity and rate capability. Modulating the MOF's composition fine-tunes its electrochemical properties, while core–nanoshell architecture provides stability and conductivity benefits. Surface functionalization with active groups promotes specific electrochemical reactions, and nano-structuring techniques optimize surface area and transport properties.

In addition to the electrode material, the energy density of the supercapacitor also depends on the choice of appropriate electrolyte because it is directly proportional to the square of the potential window. Among different electrolytes, aqueous and organic solutions have been employed as electrolytes in most SC devices. These aqueous and organic electrolytes served as a conduit for transferring charges. However, the redox reactions at the working electrode surfaces have depended on the electrochemical performance of the current generation pseudocapacitors and hybrid capacitors. Researchers have recently tried to initiate redox processes in the electrolytes 20 to improve the SC's overall performance. Redox additives and additional electrolytes have been utilized in recent years to achieve this. A K_3_[Fe(CN)_6_]-based redox additive has been widely used among the many redox additive electrolytes to improve performance.^22–24^ This specific electrolyte variant includes a minimal quantity of a redox additive, enabling direct involvement in swift electron transfer during reversible oxidation–reduction reactions at the interface between the electrode and electrolyte. This engagement *via* a redox couple notably amplifies the electrochemical performance of the supercapacitor.^25^ Previous studies have predominantly utilized aqueous electrolytes to enhance the overall capacitive properties of supercapacitors. These additives include K_3_[Fe(CN)_6_], CuSO_4_, KI, VOSO_4_, hydroquinone, naphthoquinone, and phenylenediamine.^26–28^ Notably, K_3_[Fe(CN)_6_], distinguished by its lower toxicity, exceptional stability, and reversibility, holds particular significance due to its augmentation of the faradaic capacity within an electrochemical system. This compound introduces the ferrocyanide/ferricyanide ([Fe(CN)_6_]^4−^/[Fe(CN)_6_]^3−^) redox couple, facilitating additional redox reactions.^29^ The presence of this redox couple in electrolytes diminishes key resistance parameters at the electrode surface, such as solution resistance and charge transfer resistance. This reduction is attributed to the influence of redox-active materials, contributing to an accelerated charge transfer rate. Consequently, it is acknowledged that employing suitable redox additive electrolytes holds promise for enhancing the overall electrochemical performance of supercapacitors.^30^

Therefore, in this work, we have employed a Z867 core–nanoshell composite as a potential electrode for supercapacitors, and its synergy is studied with optimized redox additive electrolyte *i.e.*, 0.2 M K_3_[Fe(CN)_6_] + 1 M Na_2_SO_4_ (RAE). A Z867 core–nanoshell electrode and redox additive electrolyte combination offers highly redox active sites and allows for efficient charge migration at the electrode–electrolyte interface, which is responsible for the remarkable performance of supercapacitors. Furthermore, Z867 was assembled as the cathode and anode of the symmetrical supercapacitor device in the presence of redox-additive-based electrolytes. Our research has shown that the Z867 redox system can produce high specific capacitance and energy density values and exceptional cycling stability compared with their pristine counterparts. Compared to previously reported MOF-based devices, the symmetrical supercapacitor assembled using Z867 electrodes has also exhibited improved energy and power density values, confirming the successful synergy strategy of a core–nanoshell MOF electrode and redox additive electrolyte.

## 2. Electrode preparation, device assembly, and electrochemical characterization of electrodes

### 2.1. Preparation of electrodes and electrochemical measurements

The experimental details for synthesizing the Z867 core–nanoshell structure and material characterization are given in Section S1 of the ESI.†^31^ The electrode is prepared by forming a uniform slurry of the active material (ZIF-67, ZIF-8, and Z867), carbon black, and polyvinylidene fluoride (PVDF) in a weight ratio of 8 : 1 : 1 respectively in NMP solvent. Furthermore, the resultant slurry was drop cast over graphite foil in an appropriate quantity, and dried for 12 hours at 80 °C in a vacuum oven. The estimated material deposition on the electrode is 1.52 mg cm^−2^. All the electrochemical measurements were performed on a PGSTAT-302N system from Autolab (Metrohm, NOVA 10.5 software), in which cyclic voltammetry (CV), galvanostatic charge–discharge (GCD), and electrochemical impedance spectroscopy (EIS) were carried out. In a three-electrode electrochemical system, Ag/AgCl served as the reference electrode, Pt as the counter electrode, and the active material (ZIF-67, ZIF-8, and Z867) on a Grafoil substrate as the working electrode. The electrochemical studies were conducted within a potential window of −0.1 to 0.5 V. Various scan rates of 100, 50, 20, 10, and 5 mV s^−1^ were applied for CV. The GCD analysis was also carried out with a cut-off potential range of −0.1 to 0.5 V (potential difference = 0.6 V) at different current densities of 4.5, 6, 7.5, and 9 A g^−1^. The EIS measurements were also conducted at a constant AC potential of 10 mV with frequencies ranging from 0.1 Hz to 100 kHz.

To fabricate the symmetric supercapacitor device, two Z867 electrodes with equal mass loading were prepared to serve as both positive and negative electrodes. A filter paper separator with a pore size of 0.45 μm was employed. Both the electrodes and separator were immersed in the redox additive electrolyte for 8 hours. Subsequently, the device was assembled by placing the separator between the electrodes and securing them with a paraffin film for support. The electrochemical performance of the device was assessed using an electrochemical workstation, model: VSP from Biologic (Germany) through cyclic voltammetry (CV), galvanostatic charge–discharge (GCD), and electrochemical impedance spectroscopy (EIS) analyses for evaluating the specific capacitance, energy density, and power density of the symmetrical SC device.

The detailed equations for calculations of electrochemical performance in three and two-electrode systems are given in Section S1.4 of the ESI (eqn (S1)–(S5)†).

## 3. Results and discussion

### 3.1. Structural and morphological characterization

**Fig. 1:**
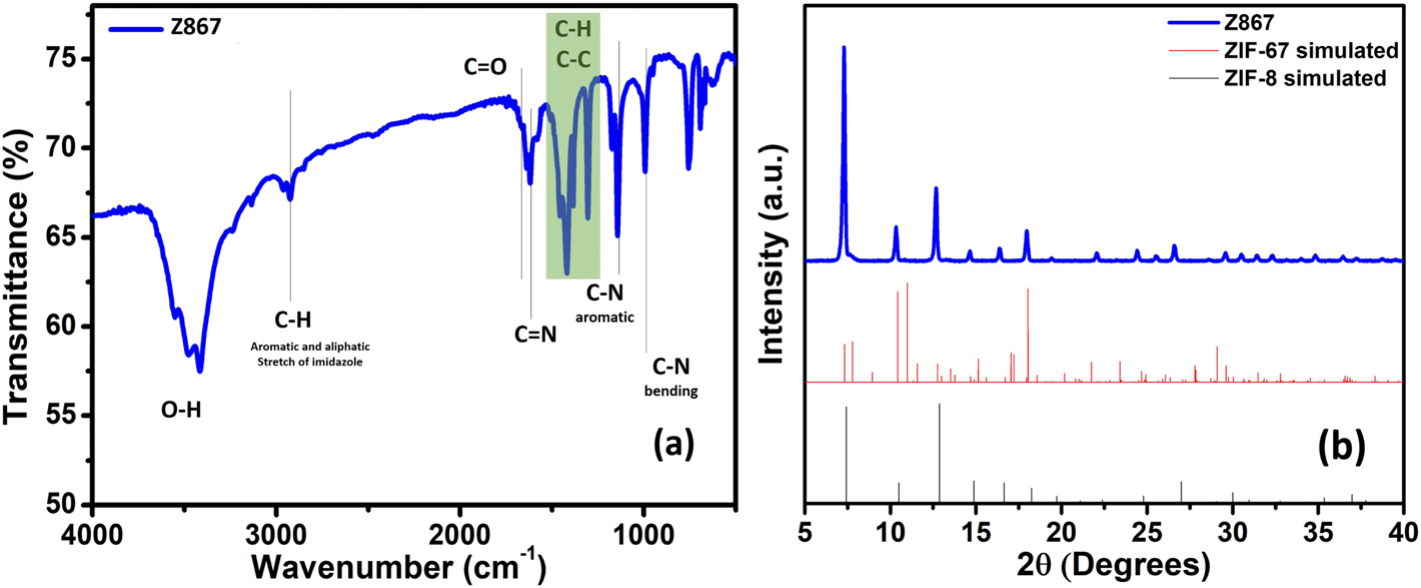
(a) FTIR spectra and (b) XRD spectra of the Z867 core–nanoshell structure.

The production of MOFs and the construction of their core–nanoshell are also validated using FTIR analysis. Fig. 1a shows the FTIR spectra of the Z867 core–nanoshell structure. FTIR analysis has been used to determine whether functional groups are present in the Z867 crystal. Due to 2-methylimidazole (2mIm) molecules, vibrational bands between 600 and 1500 cm^−1^ are observed. A band represents the stretching vibrational mode of C=N in the 2mIm structure in the sample at 1420 cm^−1^. Four distinctive peaks for the ZIF-8 particles correspond to the vibrations of the C–H, C=N, C–N_aromatic_, and C–N_bending_ in the imidazole ring at 2930, 1600, 1140, and 990 cm^−1^, respectively. For comparison, FTIR spectra of bare ZIF-8 and ZIF-67 MOF are given in Fig. S1a of the ESI.† The bare MOF FTIR signals match well with those of the core–nanoshell structure.

To analyze the structural characteristics of the Z867 core–nanoshell nanocomposite, X-ray diffraction (XRD) analysis was performed, as shown in Fig. 1b. It displayed numerous characteristic peaks at 2*θ* values of 7.2, 10.3, 12.7, 16.4, and 17.8°, indexed to the (011), (002), (112), (013), and (222) planes of ZIF-67.^32^ The fact that the ZIF-67 and Z867 diffraction peaks nearly coincide, thereby indicates that the core of ZIF-8 has little impact on the crystallization of ZIF-67. Remarkably, the XRD analysis of core–nanoshell Z867 crystals provides intriguing insights. Despite having a similar crystal structure, ZIF-8 (JCPDS 00-062-1030) and ZIF-67 (JCPDS 671073) display distinct peak intensities. XRD of ZIF-8 and ZIF-67 is also shown in Fig. S1b.† In the core–nanoshell structure, the peaks are almost identical to those of ZIF-8,^33^ suggesting a delicate interplay between the two components. This intriguing phenomenon can be attributed to the thin nanometer layer of ZIF-67 enveloping the ZIF-8 core, confirmed by HRTEM analysis (discussed later). The dominance of ZIF-8 peaks indicates that the diffraction contribution primarily stems from the ZIF-8 core, while the ZIF-67 nanoshell has a relatively minor influence. The nearly identical peak positions underscore the closely related crystallography of the two components. Moreover, in the case of ZIF-8, a tilt in the higher 2*θ* peak is observed, indicating possible lattice strain or disorder within the ZIF-8 crystals.^33^ However, no peak tilting is observed in the core–nanoshell structure, where ZIF-8 serves as the core. Interactions between the ZIF-8 core and the ZIF-67 nanoshell in the core–nanoshell structure may help mitigate this strain.^34^ Additionally, the formation of the Z867 core–nanoshell structure might involve a process that enhances the crystallinity of the core while concurrently affecting the crystallographic arrangement of the ZIF-67 nanoshell, ultimately reducing the strain in the ZIF-8 crystal.^35^

**Fig. 2:**
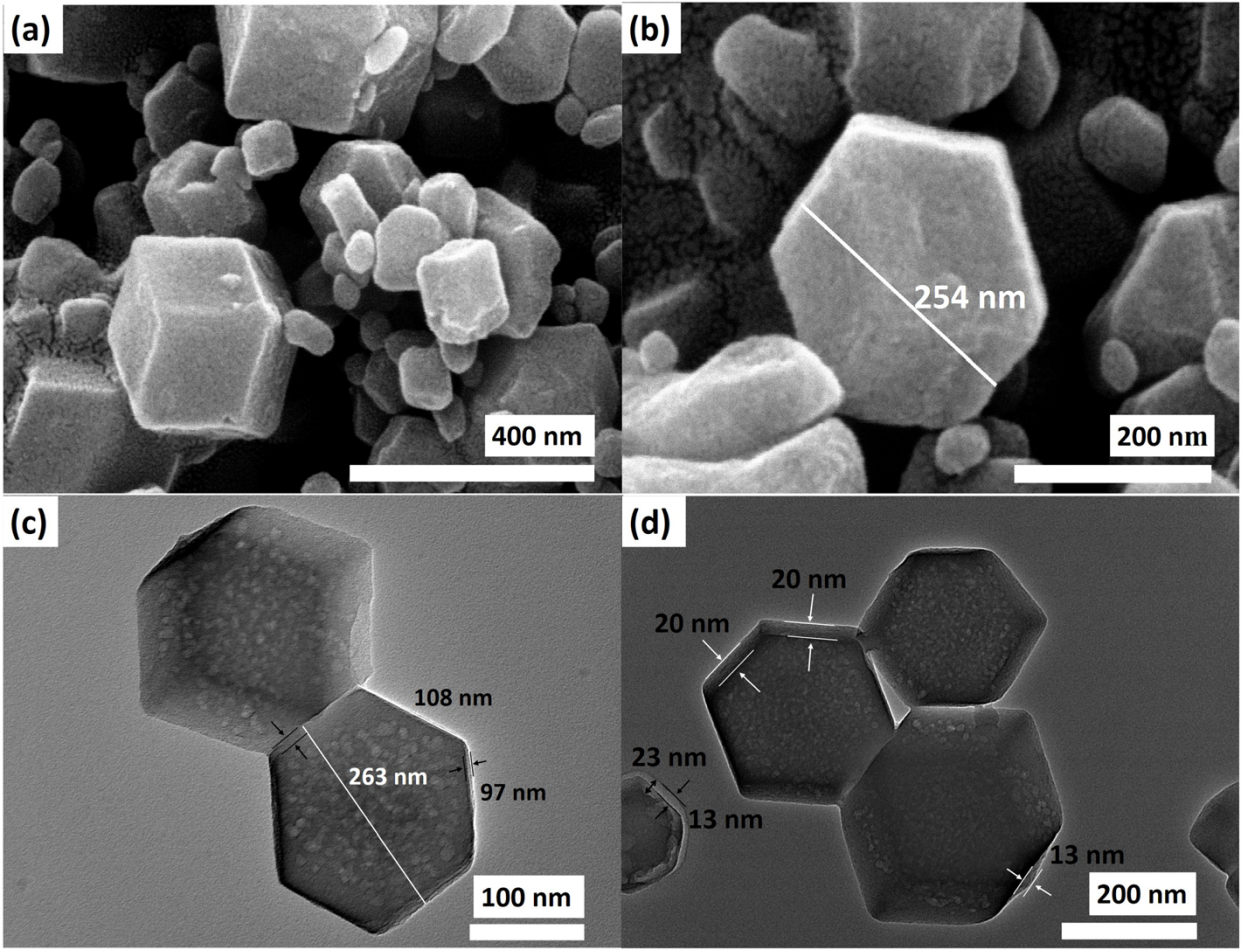
(a and b) FE-SEM images and (c and d) TEM image of the Z867 core–nanoshell structure.

The presence of imidazole and the crystal structures resembling ZIF-8 and ZIF-67 are validated through XRD and FTIR analyses, affirming the formation of the ZIF structure. Hence, to confirm the formation of the core–nanoshell structure, FE-SEM and TEM analyses were also performed to observe the morphological structure. The FE-SEM images depicted distinct rhombic dodecahedral structures of Z867, featuring a textured surface (Fig. 2a and b). The irregular surface likely signifies uneven growth of the ZIF-67 layer on the ZIF-8 structure. In some regions, the crystal structure's surface appears fractured, possibly due to the relatively easy extraction of the ZIF-67 layer in areas where the lattices of both ZIF-8 and ZIF-67 experience strain. These surface distortions can also stem from differing crystalline properties between ZIF-8 and ZIF-67 and the transition from the core's crystal lattice to that of the nanoshell. This transition may not happen uniformly across the surface, resulting in structural variations.

Moreover, the growth of ZIF-67 over ZIF-8 could induce interactions and structural rearrangements, reflecting surface irregularities. For comparison, FESEM of ZIF-8 and ZIF-67 is also shown in Fig. S2.† The crystal structures of ZIF-8 and ZIF-67 are almost similar as they are polyhedra. The same type of crystal structure can be observed for Z867. However, due to the limited magnification in the FESEM images and the challenging contrast, determining the ZIF-67 layer's thickness is difficult. To address this, we conducted a TEM analysis, revealing the rhombic dodecahedral shape of the core–nanoshell structure in more detail (Fig. 2c and d).

The layers of ZIF-67 covering the ZIF-8 crystal are distinguishable due to the contrasting appearance of the nanoshell and core (Fig. 2d). Examination of TEM images revealed non-uniform thickness of the ZIF-67 layer ranging from 20 nm to 25 nm. For instance, in the core–nanoshell structure shown in Fig. 2d, the observed thickness varies from a minimum of 20 nm to a maximum of 25 nm. Furthermore, holes in the ZIF-8 core suggest incomplete crystallization or formation during the synthesis process. These areas might have remained as voids or less dense regions within the structure, potentially arising from Ostwald ripening, where smaller crystals dissolve and contribute to the growth of larger crystals. In areas where the ZIF-8 core is less stable, has lower crystallinity, or exhibits strain (as seen in XRD), dissolution might have occurred to some extent, resulting in the formation of holes. The presence of non-uniform surface features and holes within the ZIF-8 core holds significant implications for the electrochemical properties of the core–nanoshell structure. The uneven surface may influence the effective surface area accessible to electrolyte ions, affecting capacitive behaviour and energy storage performance. The holes within the ZIF-8 core could also impact ion diffusion pathways, electron transfer, and overall conductivity.

**Fig. 3:**
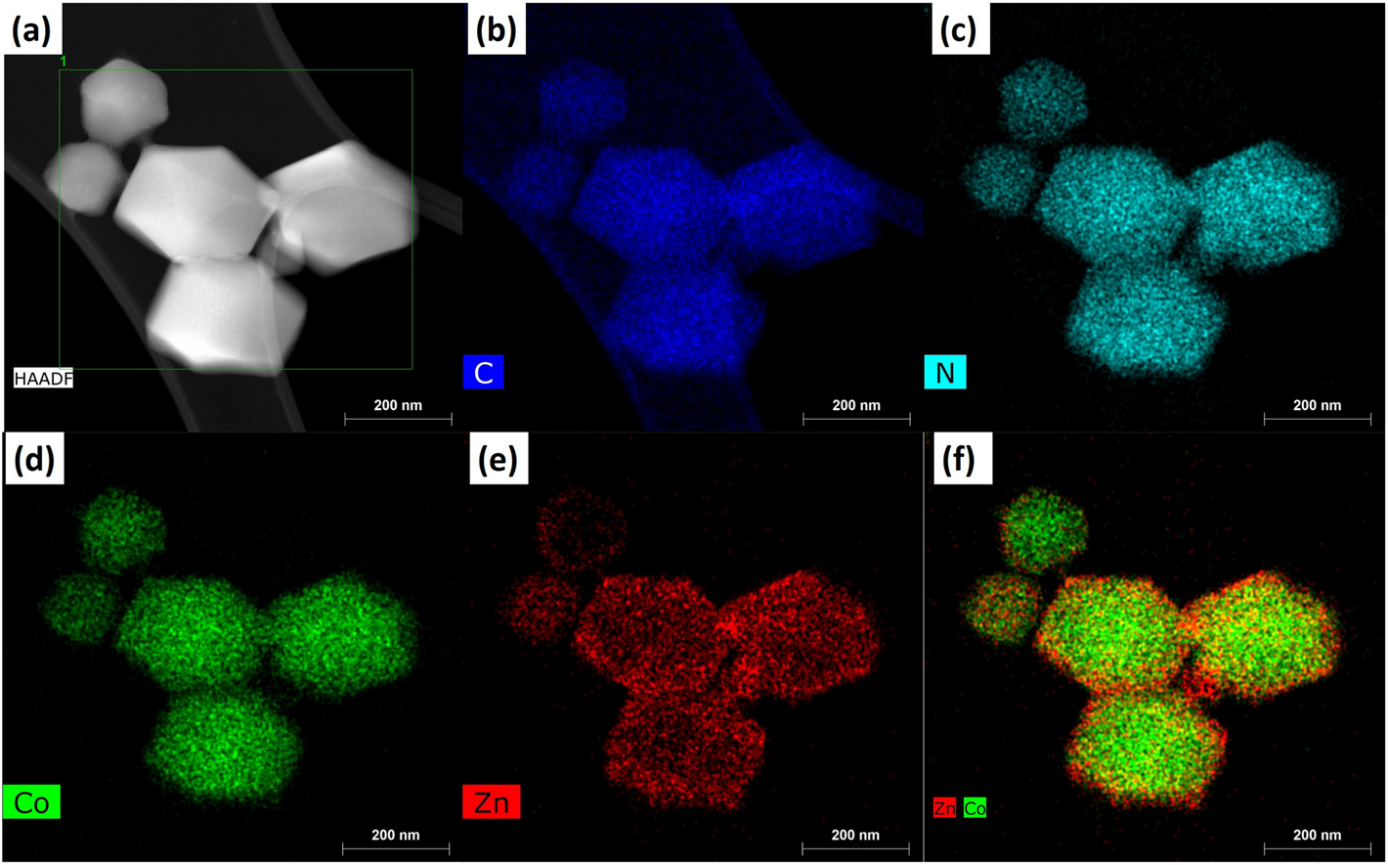
(a) HRTEM-HAADF TEM image of Z867 and the corresponding energy dispersive X-ray spectroscopy (EDXS) elemental mapping for (b) carbon, (c) nitrogen, (d) cobalt, (e) zinc, and (f) appearance of both zinc and cobalt.

Furthermore, the elemental distribution for the core–nanoshell structure is given in Fig. 3a–f. The TEM image that was used for the elemental mapping is presented in Fig. 3a. The carbon and nitrogen mapping shows the uniform distribution through the core–nanoshell structure, which is contributed by the organic linker shown in Fig. 3b and c. Furthermore, Co and Zn metal distribution is also shown in Fig. 3d and e. However, the Zn distribution seems relatively less in the center part of the core–nanoshell crystal because Zn is in the core and Co is at the surface. Since elements are deeper within the sample, the electron beam must penetrate more material, which can lead to significant signal attenuation. This makes the signal from core elements weaker. Due to this reason, the distribution of Co is relatively high in the center, but Zn has a lower number of spots in the center. The combined elemental image of Zn and Co is also given in Fig. 3f, which shows the presence of Co metal at the surface, whereas Zn is below the Co depicting the core–nanoshell structure of Z867.

**Fig. 4:**
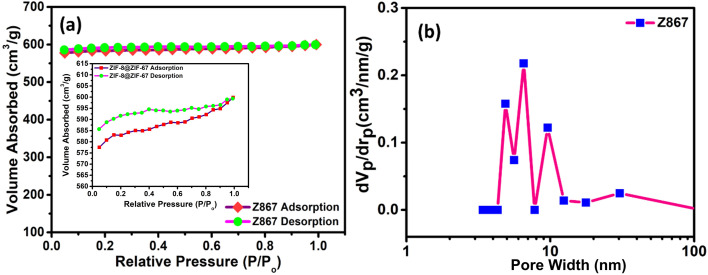
(a) BET N_2_ adsorption–desorption analysis; (b) pore size distribution curve of the Z867 core–nanoshell structure.

Subsequently, we analyzed N_2_ adsorption–desorption isotherms on the core–nanoshell Z867 composite. Our objective was to enhance its properties, focusing on achieving a high specific surface area and a well-defined pore size distribution, as depicted in Fig. 4. The investigation of N_2_ adsorption–desorption isotherms for the Z867 core–nanoshell structure uncovered a sophisticated pore network comprising macropores and mesopores. Using Brunauer–Emmett–Teller (BET) analysis to interpret the adsorption–desorption isotherm provides critical insights into the material's surface characteristics and porosity, which is fundamental for understanding its potential applications. The isotherm diagrams illustrated in Fig. 4a depict the type IV adsorption isotherms for Z867. The sharp increase in N_2_ adsorption at low pressure (*P*/*P*_o_ = 0–0.1) suggests micropores, whereas the observed hysteresis loops (inset of Fig. 4a) strongly support the presence of mesopores in the Z867 sample. Notably, the Z867 sample demonstrates significantly higher N_2_ adsorption capacities, resulting in an augmented specific surface area and pore volume. Following the initial uptake, the isotherm transitions into a linear phase, signifying adsorption on the outer surface and within mesopores.^33,34^ These mesopores, ranging from 2 to 50 nm, provide a larger surface area than micropores. The consistent linear adsorption pattern indicates a considerable presence of mesopores within the core–nanoshell structure. During the desorption phase, a hysteresis loop appears, suggesting capillary condensation within the mesopores. This occurs when gas trapped in smaller pores resists desorption, leading to a gradual release of adsorbed molecules. A hysteresis loop during desorption indicates well-connected mesopores facilitating capillary condensation.^35^ As shown in Fig. 4b, a notable presence of pore sizes within the mesoporous range was evident from the Barrett–Joyner–Halenda (BJH) pore size distribution analysis. The average pore size fell within the range of 2 to 30 nm for the Z867 material *i.e.*, 2.26 nm, which confirms the high level of mesoporosity in the core–nanoshell material.

### 3.2. Electrochemical characterization of the three electrode system

Redox active electrolytes play a crucial role in augmenting supercapacitors' electrochemical performance and functionalities. These electrolytes commonly integrate redox-active species, such as K_3_[Fe(CN)_6_] or analogous compounds, which engage in reversible oxidation–reduction reactions throughout the charge–discharge cycles of the supercapacitor. Including redox additives introduces additional redox reaction sites, augmenting the device's specific capacitance and energy storage capacity. These additives help to mitigate charge transfer limitations by facilitating rapid electron transfer at the electrode–electrolyte interface, thereby improving the overall charge/discharge kinetics. Moreover, redox additive electrolytes stabilize the electrode potential, reduce internal resistances, and enhance the device's cycling stability and long-term performance. Consequently, using redox additive electrolytes is a promising strategy to boost the efficiency, energy density, and reliability of supercapacitors for various applications, ranging from portable electronics to energy storage in renewable systems.^36,37^

Three electrode configurations were used to test the electrochemical capabilities of ZIF-8, ZIF-67, and Z867 electrodes using CV, GCD, and EIS analysis. The complete system displayed typical redox peaks in 1 M Na_2_SO_4_ + 0.2 M K_3_[Fe(CN)_6_] (RAE) when compared with the 1 M Na_2_SO_4_ aqueous electrolyte. The concentration of 0.2 M K_3_[Fe(CN)_6_] has been optimized from previous studies.^38–40^ CV has been carried out within a potential range of −0.1 to 0.5 V. The CV plot demonstrates the occurrence of faradaic charge transfer processes and the redox electrolyte K_3_[Fe(CN)_6_] within the solution. The likely reaction mechanism involved in this process is shown below:

1 [Fe(CN)_6_]^3−^ + e^−^ ⇌ [Fe(CN)_6_]^4−^

Despite increasing the scan rate, the shape of the CV curves remains relatively consistent, indicating the impressive rate capability of the Z867 electrode. This stability suggests the electrode's capability for swift faradaic redox reactions even at higher scan rates.^41,42^

**Fig. 5:**
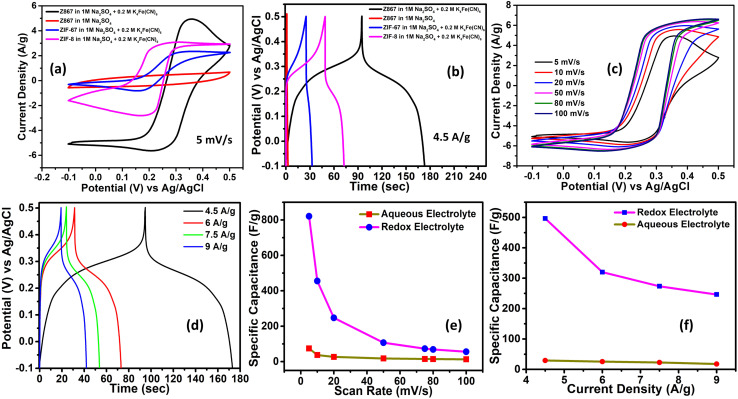
Electrochemical performance comparison of the core–nanoshell Z867 electrode material (in aqueous electrolyte and RAE) with pristine ZIF-8 and ZIF-67 in RAE electrolytes: (a) comparison between CV plots at 5 mV s^−1^, (b) comparison between GCD plots at 4.5 A g^−1^, (c) CV curves recorded at different scan rates in RAE for the Z867 electrode, (d) GCD plots recorded at different current densities in RAE for the Z867 electrode, (e) comparison of variations in the specific capacitance values at different scan rates in aqueous electrolyte and RAE for the Z867 electrode; (f) comparison of variation in the specific capacitance values at different current densities in aqueous electrolyte and RAE for the Z867 electrode.

Using the CV method, the electrochemical characteristics of supercapacitor electrodes have been investigated in terms of specific capacitance. A comparison of CV and GCD for pristine ZIF-67 and ZIF-8 in RAE and 1 M Na_2_SO_4_ electrolyte is also shown in Fig. S3 of the ESI.† The higher CV area and larger discharge time in RAE compared to 1 M Na_2_SO_4_ directly demonstrate the superiority of the redox electrolyte. Fig. 5a shows the CV comparison of the Z867 electrode, bare ZIF-8, and ZIF-67 at a scan rate of 5 mV s^−1^ using RAE. Z867 CV (black curve) in RAE had the highest peak current and the largest CV area when compared to Z867 (red curve) in aqueous electrolyte. The complete study of CV for Z867 in 1 M Na_2_SO_4_ is given in Fig. S4.† No redox peaks have been observed in the CV curve.

Furthermore, the CV of bare ZIF-8 and ZIF-67 also shows a lower CV area than that of Z867, probably due to the synergy between ZIF-67 and ZIF-8 material. The core–nanoshell material showed a specific capacitance of 820 F g^−1^ compared to 210 F g^−1^ for ZIF-67 and 434 F g^−1^ for ZIF-8 at a scan rate of 5 mV s^−1^. This demonstrates that the core–nanoshell construction of the Z867 electrode will produce a substantially higher specific capacitance. Conversely, slower scan rates are expected to enhance specific capacitance values by providing sufficient time for electrolyte ions to undergo adsorption/desorption at the electrode interface. The area integrated under the curve is notably larger in the redox electrolyte compared to the aqueous one, leading to a more pronounced performance in the former. The huge difference in specific capacitance for Z867 in the two electrolytes is due to redox activity of the RAE. The RAE introduces additional redox-active species into the system, enabling faradaic reactions at the electrode–electrolyte interface. These electrolytes are well reported in the literature to enhance the faradaic contribution in the charge storage. However, the main limitation of the redox electrolyte is the shuttling effect which leads to a lower coulombic efficiency. But the micropores in the core and the meso–macropores at the shell allow efficient access to the electrochemically active sites and inhibit the shuttling of redox species. The redox reactions significantly enhance the charge storage capacity by contributing to pseudocapacitance. In contrast, 1 M Na_2_SO_4_ is a neutral aqueous electrolyte that primarily relies on electric double-layer capacitance (EDLC). It does not involve faradaic redox reactions, leading to much lower specific capacitance (134.7 F g^−1^).^43,44^

Additionally, higher scan rates led to an increased separation between the redox peaks, which can be attributed to a diffusion-limited redox process.^45^ The specific capacitance using CV curves was calculated using eqn (S1) of the ESI.†Fig. 5b compares the GCD curves for the Z867, ZIF-8, and ZIF-67 electrodes in RAE and aqueous electrolyte at a constant current density of 4.5 A g^−1^. The Z867 electrode in RAE performed better than the Z867 electrode in aqueous electrolyte as well as bare ZIF-8 and ZIF-67 in RAE, according to the results obtained from CV analysis, which once again amply supported the superiority of using RAE. The existence of quasi-reversible faradaic reactions led to the observation of non-linear GCD patterns. The charge/voltage ratio is not constant and changes over time as a result of various faradaic reactions. The discharging current area was integrated to determine the values of individual capacitances in the systems stated above (using eqn (S2)†). At a current density of 4.5 A g^−1^, the value of specific capacitance with Z867 electrodes has been calculated to be 496.4 F g^−1^ in RAE which is much higher than that in aqueous electrolyte. A maximum specific capacitance of 29.45 F g^−1^ at 4.5 A g^−1^ current density was observed in 1 M Na_2_SO_4_ electrolyte, which is much lower than that in RAE. For comparison, ZIF-8 and ZIF-67 delivered a specific capacitance of 194 F g^−1^ and 50 F g^−1^ only in RAE which is much lower than that of the Z867 core–nanoshell electrode under similar conditions. The establishment of a core–nanoshell structure has facilitated faster electron transport and improved the efficient diffusion of ions into the redox sites of the composite material. This accounts for the higher specific capacitance observed in the Z867 electrode. Fig. 5c displays the cyclic voltammograms that were obtained when the Z867 electrodes were operated in RAE at different scan rates (100, 80, 50, 20, 10, and 5 mV s^−1^) and in different potential ranges between −0.1 and +0.5 V. Due to the faradaic character of the ZIF-67 component, the Z867 electrode features visible anodic and cathodic peaks. The values of specific capacitances (*C*_s_, in F g^−1^) were estimated using CV data. Adequate time for electrolyte ions to undergo adsorption/desorption at the electrode interface implies that utilizing lower scan rates should yield superior specific capacitance values. According to the aforementioned comparison investigation, the Z867 electrode would provide significantly larger current values in the presence of RAE. The use of RAE was unquestionably advantageous in getting a significantly higher value of specific capacitance than the pristine aqueous electrolyte without any redox additives. For instance, Z867 produced a high value of specific capacitance (820 F g^−1^ at 5 mV s^−1^) with the use of the RAE with a fixed scan rate of 5 mV s^−1^ as opposed to a value of 134.7 F g^−1^ with the 1 M Na_2_SO_4_ aqueous electrolyte.

A supercapacitor system's GCD analysis also enables us to evaluate several significant electrochemical properties, such as specific capacitance at different current densities. Fig. 5d shows the findings of GCD tests conducted on the Z867 electrode with RAE at various current densities. The Z867 electrode produced more significant electrochemical activity at lower current densities. The GCD profiles showed non-linear characteristics with a minimal voltage drop, which suggested that the electrode had a low internal resistance. Additionally, an increase in current density resulted in a lower discharge time. The previously noted observation can be linked to a rapid (yet incomplete) diffusion of electrolyte ions into the pores of the electrode material at high current densities. The cyclic voltammograms of the Z867 electrode have a greater integrated area under the curve in RAE as compared to the aqueous electrolyte (fixed scan rate), indicating a higher specific capacity. The highest specific capacitance of the Z867 electrode was determined to be 820 F g^−1^ at a scan rate of 5 mV s^−1^. According to Fig. 5e, the specific capacitance value decreases consistently as the scan rate increases. The decrease in specific capacitance is due to the electrolyte ions' failure to uniformly or completely enter the pores of the electrode material at rapid scan speeds. Fast scan rates cause partial redox reactions at the electrode surface, which are detrimental to the adsorption of electrolytes. Either of the aforementioned arguments can account for a drop in specific capacitance. Fig. 5f shows the values of specific capacitance as a function of current density, demonstrating that a low current density promotes improved specific capacitance. This feature is explained by the fact that electrolyte ions have more time to enter the pores of the active electrode surface at lower current densities, which in turn promotes more effective electronic and ionic transport.^46,47^

After CV and GCD characterization, it can be concluded that the higher performance of Z867 can be attributed to the synergistic effect of both materials *i.e.* ZIF-8 and ZIF-67. The first thing to note is the CV curves. In the presence of the same concentration of RAE, Z867 is showing much higher redox activity for the reversible redox reaction for ferricyanide. The higher redox activity of ferricyanide on the same physical area clearly indicates the higher number of electrochemically active sites which are absent in pristine ZIF-8 and ZIF-67. The ZIF-8@ZIF-67 (Z867) composite benefits from the synergistic interaction between ZIF-8 (zinc-based) and ZIF-67 (cobalt-based) components. During the charging/discharging process, these components play complementary roles, resulting in enhanced electrochemical performance. The combination of ZIF-8 and ZIF-67 results in a hierarchical porous structure, with micropores from ZIF-8 and mesopores/macropores contributed by ZIF-67. The mesopores and macropores of the ZIF-67 allow the bulk penetration of electrolyte whereas micropores of ZIF-8 are well known for the maximum charge storage (but they lack the flawless penetration of electrolyte for the bulk utilization of the material). Therefore, the hierarchical porosity created by the ZIF-8 core and ZIF-67 shell facilitates rapid ion diffusion. The same can also be seen in the GCD curves where the coulombic efficiency is better for Z867. Due to inefficient porosity in bare ZIF-67 and ZIF-8, the shuttling of the redox couple leads to a lower coulombic efficiency.

**Fig. 6:**
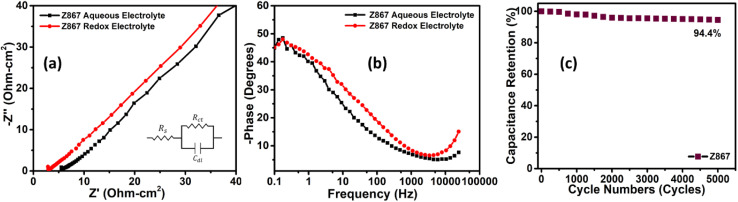
(a) Nyquist plot, (b) phase *vs.* frequency Bode plot for Z867 in aqueous and RAE electrolytes, and (c) cycling stability test for the Z867 core–nanoshell electrode material.

The electron transport and ion diffusion resistance of Z867 electrodes wesre evaluated using EIS measurements spanning from 10 mHz to 100 kHz. The Nyquist plot of Z867 exhibited nearly straight lines in the low-frequency region and semi-circular arcs in the high-frequency region, as illustrated in Fig. 6a. The intersection of the semicircle and the *Z*′-axis in the high-frequency spectrum represents the equivalent series resistance (*R*_s_), encompassing the internal electrode resistance, interface resistance between the electrode material and the current collector, and the electrolyte resistance.^48,49^ Meanwhile, the diameter of the semicircle in the high-frequency region denotes the charge transfer resistance (*R*_ct_). Observations revealed that Z867 displayed remarkably low values for *R*_s_ in RAE *i.e.*, 2.96 ohm cm^2^, which is lower than that of the Z867 electrode in aqueous electrolyte (5.68 ohm cm^2^). The absence of a prominent semi-circular region in the Nyquist plot signifies an almost negligible charge transfer resistance (*R*_ct_), indicating minimal impedance experienced at the interface between the electrode and the electrolyte. Upon comparing Nyquist plots for the redox-active and aqueous electrolytes, both exhibited similar behavior, confirming consistent electrochemical kinetics. Since the EIS measurements were performed at the open-circuit potential (OCP), no redox reactions are expected as these typically occur within the voltage range of 0.15–0.45 V *vs.* Ag/AgCl, indicated by the redox peaks in the CV curves. Therefore, Nyquist plots for both electrolytes showed identical behavior, as no redox reactions were occurred. However, a slight decrease in solution resistance was observed in the redox-active electrolyte, suggesting an enhancement in the ionic conductivity within the RAE.

The phase *versus* frequency Bode plot showcases the phase shift between the input and output signals in a logarithmic frequency scale (Fig. 6b). As the frequency decreases, the phase angle gradually deviates from zero towards 45 degrees, depicting the ion diffusion limitations. The observed phase angle of around 45 degrees for the Z867 electrode material in 1 M Na_2_SO_4_ suggests a capacitive-dominant behaviour. The frequency at which the phase angle for Z867 in RAE is around 45 degrees is often referred to as the crossover frequency. It represents the point where the capacitive and resistive elements in the supercapacitor contribute equally to the overall impedance, resulting in a 45-degree phase shift.

In order to assess the practical utility of the electrode material, the cycling stability of the Z867 electrode was examined. The Z867 electrode underwent repetitive charge–discharge cycles, amounting to 5000 cycles at a current density of 20 A g^−1^ (Fig. 6c). Post-stability testing showed that Z867 retained 94.4% of its initial capacitance, indicating its exceptional stability. This high retention percentage underscores the stability and reliability of the electrode material under repeated cycling. Furthermore, to analyze any structural changes before and after cycling, we performed the SEM and EDS characterization as shown in Fig. S5 of the ESI.† The SEM image of the working electrode before cycling shows the crystal of CSMOF covered with carbon black and PVDF, which is used as a conductive agent and binder, as shown in Fig. S5a.† The EDS spectra show the elements in the electrode material, mainly including carbon, oxygen, nitrogen, cobalt, and zinc (Fig. S5b†). The presence of Au is from the gold coating through sputtering before the SEM analysis to reduce any charging at the surface. After cycling, SEM and EDS are also shown in Fig. S5c and d.† The SEM image shows negligible changes in the crystal structure of CSMOF. Like before cycling, the crystals are covered with carbon black and PVDF (Fig. S5c†). However, the EDS of the electrode material shows the presence of potassium as well as iron from the electrolyte solution (Fig. S5d†). This confirms the redox SC device's superior stability and outstanding efficiency over the long run. The stable performance at a high current density of 20 A g^−1^ is noteworthy, as it demonstrates the electrode material's ability to withstand elevated charge–discharge rates, which is critical for applications requiring rapid energy storage and release. However, 6% of capacitance decay is probably due to potential mechanisms, including electrode material degradation due to repeated ion insertion/extraction processes, electrolyte decomposition leading to unwanted by-products, and sluggish ion intercalation/deintercalation kinetics with time.

### 3.3. Electrochemical performance of the redox symmetrical supercapacitor device

**Fig. 7:**
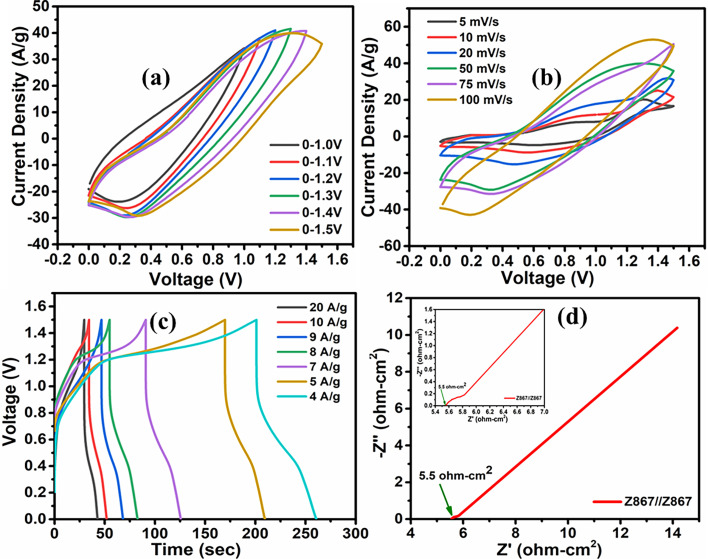
Electrochemical performance of the Z867//Z867 redox symmetrical supercapacitor device. (a) Voltage window test, (b) CV curves at different scan rates, (c) GCD curves at various current densities, and (d) Nyquist plot (inset shows the Randles equivalent circuit and magnified Nyquist plot).

The genuine nature of an electrode becomes apparent only through constructing a supercapacitor device using it. We constructed a symmetric supercapacitor device labeled Z867//Z867 using a redox additive electrolyte in this context. Upon assembling the device, the initial step involved determining the most effective potential range for its optimal performance. To accomplish this, we conducted cyclic voltammetry (CV) across various potential windows (as shown in Fig. 7a). The findings revealed that the device performs best within the 0.0–1.5 V range, with the charge storage capacity diminishing beyond this potential window. This suggests that this specific range is favourable for achieving the highest charge storage capacity and electrochemical stability for the studied device. Beyond the 1.5 V limit, the diminishing charge storage capacity through the contraction of CV area suggests a reduction in the electrochemical stability of the system. Therefore, the 0.0–1.5 V range aligns well with the optimal voltage region for achieving higher capacitance, suggesting that the electrode material is more effective in storing charge within this window.

The CV curve's non-rectangular shape (Fig. 7b) confirms that the symmetric supercapacitor device exhibits pseudocapacitive traits. Across scan rates ranging from 5 to 100 mV s^−1^, the consistent shape of the CV plots implies reversible electrochemical activity. The CV curves indicate that the supercapacitor's charge–discharge behavior is not purely capacitive. Unlike ideal double-layer capacitors exhibiting rectangular CV shapes, pseudocapacitors involve faradaic redox reactions at the electrode–electrolyte interface, leading to non-rectangular curves. This signifies the presence of pseudocapacitive traits in the symmetric supercapacitor device. The consistent shape across various scan rates further implies reversible electrochemical activity, showcasing the suitability of the electrode material for energy storage applications with enhanced capacitive behaviour. Fig. 7c illustrates the GCD curve for the Z867//Z867 redox SC device, showcasing current densities from 4 to 20 A g^−1^. At specific current densities of 4, 5, and 9 A g^−1^, exceptional specific capacitance values of 635.2, 529.3, and 516.6 F g^−1^ were achieved. The ability of the supercapacitor to maintain high specific capacitance values at relatively high current densities (up to 9 A g^−1^) indicates good rate capability. The achieved high specific capacitance values at lower current densities (4 A g^−1^) suggest that the electrode material can effectively store charge with minimal voltage drop, indicating excellent energy storage capabilities. The exceptional specific capacitance values could be attributed to the material's porosity, which functions as an ion reservoir, facilitating swift ion transport. The collaborative characteristics of ZIF-8 and ZIF-67 in the Z867 composite likely contribute to the observed high performance.

The Nyquist plot featured in Fig. 7d displays the impedance characteristics of the Z867//Z867 redox SC device, in which the equivalent series resistance (ESR) of the device was determined to be 5.5 ohm cm^2^ based on the actual axis intercept. This observation indicates that the Z867//Z867 device encounters minimal resistance within the redox electrolyte, suggesting rapid charge transport kinetics. Consequently, these results solidify the conclusion that the Z867 material in the redox electrolyte forms a compelling combination, paving the way for a high-performance supercapacitor. The observed ESR value of 5.5 ohm cm^2^ suggests that the Z867//Z867 redox symmetric supercapacitor device encounters minimal resistance during charge and discharge processes. A lower ESR value indicates more efficient charge transport kinetics.

**Fig. 8:**
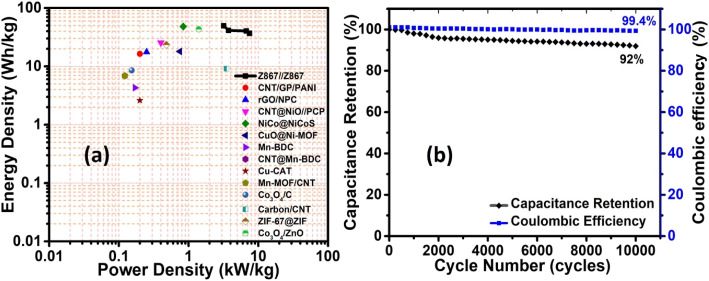
Electrochemical performance of the Z867//Z867 redox symmetrical supercapacitor device: (a) Ragone plot, and (b) cycling stability and coulombic efficiency.

Fig. 8a exhibits the Ragone plot of the Z867//Z867 redox SC device. This device demonstrates an outstanding energy density of 49.6 W h kg^−1^ at a power density of 3.2 kW kg^−1^. It maintains a high energy density of 36.7 W h kg^−1^ even at a high-power density of 7.5 kW kg^−1^. Table 1 provides a summary comparing the performance with previously reported literature. It is visible that the Z867 electrode material showed superior performance to several different MOF-based structures for supercapacitor applications. The trend in the specific capacitance of the Z867//Z867 redox SC device (Fig. S6†) emphasizes an inverse relationship. Additionally, Fig. 8b illustrates the cycling stability and coulombic efficiency of the symmetrical device, which is 92% and 99.4% respectively after 10 000 charge–discharge cycles. However, for the commercialization of the product, many fabrication steps affect the stability which needs to perform in future and can lead to another publication with dedicated stability based research.^50,51^

**Table 1:** Electrochemical performance comparison of the Z867 electrode material with previously reported electrode materials for supercapacitor application

S. no.	Material	3-Electrode setup		2-Electrode setup			Ref.
		Electrolyte	Specific capacitance (F g^−1^) (current density (A g^−1^))	Energy density (W h kg^−1^)	Power density (kW kg^−1^)	Cyclic stability	
1	CNT/GP/PANI	1 M H_2_SO_4_	500 (1)	16.4	0.2	91 (20 000)	52
2	MOF derived rGO/NPC	6 M KOH	334 (5 mV s^−1^)	17.6	0.250	97 (10 000)	53
3	Co_9_S_8_@N–C@MoS_2_ derived from ZIF	3 M KOH	340.7 (1)	—	—	101 (20 000)	54
4	CNT@NiO//PCP derived from Zn-MOFs	1 M KOH	245 (1)	25.4	0.4	93 (10 000)	55
5	MOF derived NiCo-alloy@NiCo-sulfide	3 M KOH	213 (1)	48.2	0.84	83.5	56
6	CuO@Ni-MOF	3 M KOH	474.68 (2)	18	0.75	94	57
7	Mn-BDC MOF	1 M Na_2_So_4_	177.9 (0.5)	4.3	0.17	98	58
8	CNTs@Mn-MOF	1 M Na_2_SO_4_	203.1 (1)	6.9	0.122	88	59
9	Conductive MOF nanoarray (Cu-CAT)	PVA/KCl	120 (0.5)	2.6	0.2	80	60
10	Mn-MOF/CNT	1 M Na_2_SO_4_	202.8 (1)	6.9	0.1226	88 (3000)	61
11	Co-MOF derived Co_3_O_4_/C	PVA–KOH	61.5 (0.58)	8.54	0.153	78.3 (5000)	62
12	MOF-199 derived NPC/CNT	6 M KOH	194.8 (2)	9.1	3.5	95 (10 000)	63
13	ZIF-67@amorphous ZIF	6 M KOH	1176.81 (1)	23.4	0.48	98 (1000)	64
14	Co_3_O_4_/ZnO derived from ZIF-8@ZIF-67	6 M KOH	415 (0.5)	43.2	1.4	93 (1000)	65
**15**	**Z867//Z867 redox symmetrical supercapacitor**	**0.2 M K**_**3**_ **[Fe(CN)**_**6**_ **] in 1 M Na**_**2**_ **SO**_**4**_ **(RAE)**	**496.4 (4.5)**	**49.6**	**3.2**	**92 (10 000)**	**This work**

## 4. Conclusion

In conclusion, we demonstrated the use of 0.2 M K_3_[Fe(CN)_6_] in 1 M Na_2_SO_4_ redox active electrolyte with the ZIF-8@ZIF-67 (Z867) core–nanoshell electrode for symmetrical supercapacitors. The occurrence of a redox couple provides additional redox activity, which enhances the charge transport behaviour while the porous Z867 electrode exhibits efficient charge storage. Furthermore, the Z867//Z867 device showcased remarkable electrochemical performance, achieving a high energy density of 49.6 W h kg^−1^ and a noteworthy high-power density of 3.2 kW kg^−1^. This excellent electrochemical performance of the Z867//Z867 redox supercapacitor device suggests the importance of the K_3_[Fe(CN)_6_] additive with enhanced redox reactions for high performance core–nanoshell MOF based energy storage devices. Hence, this study emphasizes the importance of careful electrode material design and demonstrates the enhancement achievable through the integration of a tailored redox additive electrolyte, thereby advancing the practical applications of supercapacitors in diverse fields.

## Data availability

Data will be made available on request from corresponding authors.

## Author contributions

Mansi performed all the synthesis, performed material and electrode studies, and wrote the manuscript; P. D. conducted redox device analysis and discussion; V. S. did manuscript cowriting, electrochemical data analysis, and discussion; A. B. contributed to detailed electrochemical analysis results, structural change analysis after cycling, and discussion on elemental mapping of the core–nanoshell structure; S. S. performed conceptualization, data analysis, discussion, and manuscript review and editing; U. K. T. did supervision of the project, manuscript review, editing, and discussion; A. D. did supervision of the project, conceptualization, manuscript review, editing and discussion.

## Conflicts of interest

All authors declare no conflict of interest.

## Supplementary Material

NA-OLF-D4NA00805G-s001
