# Supplementary material for: High performance supercapacitors driven by the synergy of a redox-active electrolyte and core–nanoshell zeolitic imidazolate frameworks
Source: Nanoscale Adv. 2025 Feb 10;7(7):2105–18. doi: 10.1039/d4na00805g (PMC11844434; doi:10.1039/d4na00805g)
Supplement: NA-007-D4NA00805G-s001 [file NA-007-D4NA00805G-s001.pdf]

Supplementary Information (SI) for Nanoscale Advances.
This journal is © The Royal Society of Chemistry 2025

# High performance supercapacitor driven by the synergy of redox-active electrolyte and core-nanoshell zeolitic imidazolate frameworks

Mansi[1,2,3], Vishal Shrivastav[3], Prashant Dubey[4], Aristides Bakandritsos[3,5], Shashank Sundriyal[3*], Umesh K. Tiwari[1,2*], and Akash Deep[2,6*]

[1]CSIR-Central Scientific Instrument Organisation (CSIR-CSIO), Chandigarh 160030, India;

[2]Academy of Scientific and Innovative Research, Ghaziabad, 201002, India;

[3]Regional Center of Advanced Technologies and Materials, The Czech Advanced Technology and Research Institute (CATRIN), Palacký University Olomouc, Šlechtitelů 27, 779 00 Olomouc, Czech Republic;

[4]Advanced Carbon Products and Metrology Department, CSIR-National Physical Laboratory (CSIR-NPL), New Delhi 110012, India;

[5]Nanotechnology Centre, Centre for Energy and Environmental Technologies, VŠB–Technical University of Ostrava, 17. listopadu 2172/15, 708 00 Ostrava-Poruba, Czech Republic;

[6]Institute of Nano Science and Technology (INST), Sector-81, Mohali, 140306 Punjab, India

*Correspondence: akashdeep@inst.ac.in (**Dr. Akash Deep**), umeshtiwari@csio.res.in (**Dr. Umesh K. Tiwari**), shashank.sundriyal@upol.cz (**Dr. Shashank Sundriyal**)

## S1. Experimental-

### 1.1 Materials

The materials utilized in this experiment were all of analytical grade purity and were not subjected to any additional processing. Sigma Aldrich provided N-methyl-2-pyrrolidone (NMP) and cobalt nitrate hexahydrate. 2-Methylimidazole was provided by Alfa Aesar. We purchased methanol, sodium sulfate($Na_2SO_4$), potassium ferricyanide ($K_3[Fe(CN)_6]$) and zinc nitrate hexahydrate ($Zn(NO)_3.6H_2O$) from Merck in India. The carbon black and polyvinylidene fluoride (PVDF) were provided by Himedia in India.

### 1.2 Synthesis of Z867 core-nanoshell composite

The synthesis process of ZIF-8 and ZIF-67 has been followed from literature [1,2]. ZIF-8 particles (80 mg) were thoroughly mixed with 10 ml of methanolic solution to create solution A, which

was then ultrasonically processed for 30 minutes to create ZIF-8@ZIF-67. After 20 minutes of magnetic stirring, a cobalt nitrate hexahydrate solution (177 mg, 3 ml) was simultaneously made. Here, MeIm's methanolic solution (895 mg in 3 ml) was gradually and progressively added, agitated for 5 minutes to thoroughly mix, and labelled solution B. Then, under gentle stirring, the two solutions A and B are thoroughly combined before being placed in an autoclave to be heated to 110°C for 12 hours. Crystals of Z867 were produced in this way. Once, the resultant samples were cooled at room temperature, it is then centrifuged and washed several times with methanol and then kept in vacuum oven at 80$^{o}$C for drying.

### 1.3 Characterization of material

The $N_2$ adsorption-desorption cycle and BJH pore size distribution of the core-nanoshell MOF are being monitored by a QuantachromeAsiQwin device. Using a field emission scanning electron microscope (FESEM, Hitachi S4800, applied voltage of 5-7 kV) and a high-resolution transmission electron microscope (HRTEM, Technai G20, accelerating voltage of 200 kV), the morphological structure of the material was investigated. For the morphological analysis before and after the cycling stability test, the electrode sample was placed onto a dual-sided carbon tape, fixed to a holder and coated by a 20 nm gold thin film, was examined by SEM using a JSM-7900F Jeol scanning electron microscope with an accelerating voltage of 10.0 kV. Energy Dispersive Spectrometry (EDS) was performed to verify the composition of the sample using Jeol JED-2300 SDD detector, with acquisition time of 60 second and under an accelerating voltage of 15.0 kV. High-resolution transmission electron microscopy (HR-TEM) in high-angle annular dark-field (HAADF) mode for elemental mapping were performed with an FEI TITAN G2 60-300 HRTEM microscope with an X-FEG type emission gun, operating at 300 kV, objective-lens image spherical aberration corrector, and ChemiSTEM energy-dispersive X-ray spectroscopy (EDS) detector. Using a Nicolet iS10 spectrometer,

measurements for Fourier transform infrared (FTIR) spectroscopy were made. The X-ray diffractometer, XRD, Bruker, D8 Advance, = 1.54 Å was used to measure the XRD patterns.

### 1.4 Electrochemical Calculations

The specific capacitance from CV and GCD curves in three electrode systems are computed using Eq. S1 and S2 respectively, where Cs is specific capacitance, Im is the current density indicating the maximum charge/discharge rate, Vdt is the Integral of voltage with respect to time, representing the discharge process over time, ΔV is potential window.

$$Cs = \frac{\int I\,(V).\,dV}{m.\,v.\,\Delta V} \tag{S1}$$

To address the non-linear GCD behavior resulting from quasi-reversible faradaic reactions, it's essential to consider that the charge/voltage ratio no longer remains constant, varying with time. Consequently, the calculation of specific capacitances for such non-linear GCD curves necessitates an adjusted approach. Therefore, the specific capacitances for non-linear GCD curves were determined using the following equation[3]:

$$Cs = 2.Im \int V\,dt \big/ V^2 \tag{S2}$$

The specific capacitance of the symmetrical supercapacitor device was calculated using Eq. S3, where C represents specific capacitance (F/g), Im denotes current density (A/g), Δt signifies the discharging time (s), and ΔV indicates the voltage window (V)[3].

$$C = 4.\ Im \times \Delta t/\Delta V \tag{S3}$$

$$E = \frac{1}{7.2}\frac{1}{4}CV^2 \tag{S4}$$

$$P = \frac{3600\,E}{t} \tag{S5}$$

For evaluating the energy density (E) in watt-hours per kilogram (Wh/kg), Equation S4 was utilized. Here, V stands for potential, and C refers to the specific capacitance. Furthermore, the power density (P) in watts per kilogram (W/kg) was determined using Equation S5, where E represents the energy density in watt-hours per kilogram (Wh/kg), and t represents discharge time in seconds.

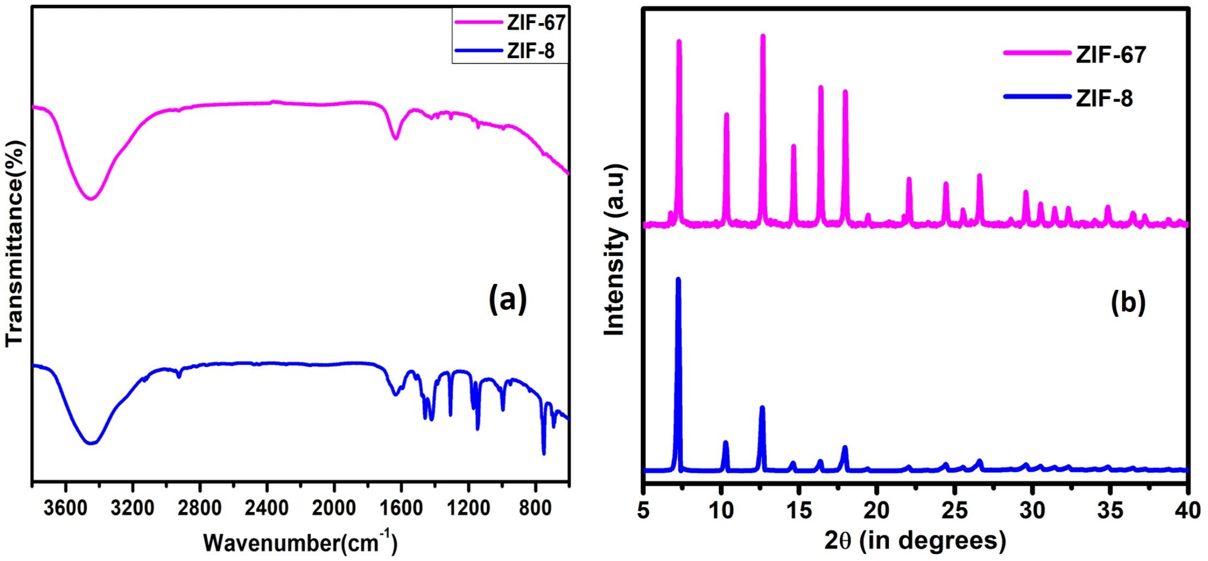


**Figure S1.** (a) FTIR of ZIF-8 and ZIF-67, (b) XRD of ZIF-8 and ZIF-67.

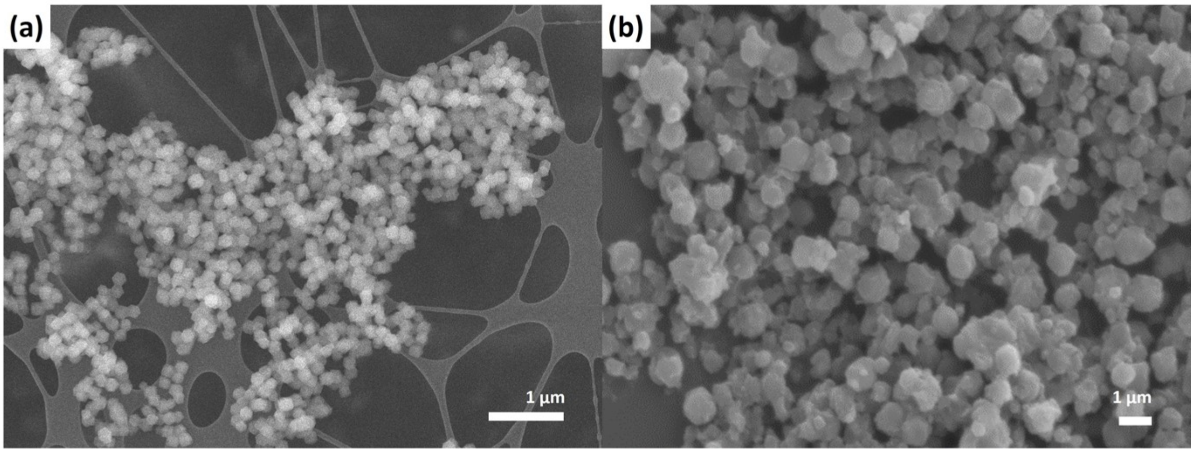


**Figure S2:** FESEM image of (a) ZIF-8 and (b) ZIF-67.

**S2. Electrochemical performance in three-electrode configuration using aqueous electrolyte**

The electrode material Z867 was examined in a 1M $Na_2SO_4$ aqueous electrolyte. At a high scan rate of 100 mV/s, the integrated area of the CV curve for the Z867 electrode increases while still retaining a slightly deviated rectangular shape. This phenomenon could be attributed to the reversibility of the reaction associated with structural stability occurring on the material's surface or on the efficiency of charge transport during the charge-discharge process **(Figure S3(a)).** The variation of specific capacitance with current density is shown in **Figure S3(b).** A maximum specific capacitance of 29.45 F/g at 4.5A/g current density was observed which is much lesser when compared with that in RAE. Besides this, the Nyquist plot gave the value of equivalent series resistance (ESR) of 4.17 ohm-cm$^2$ **(Figure S3(c)).**

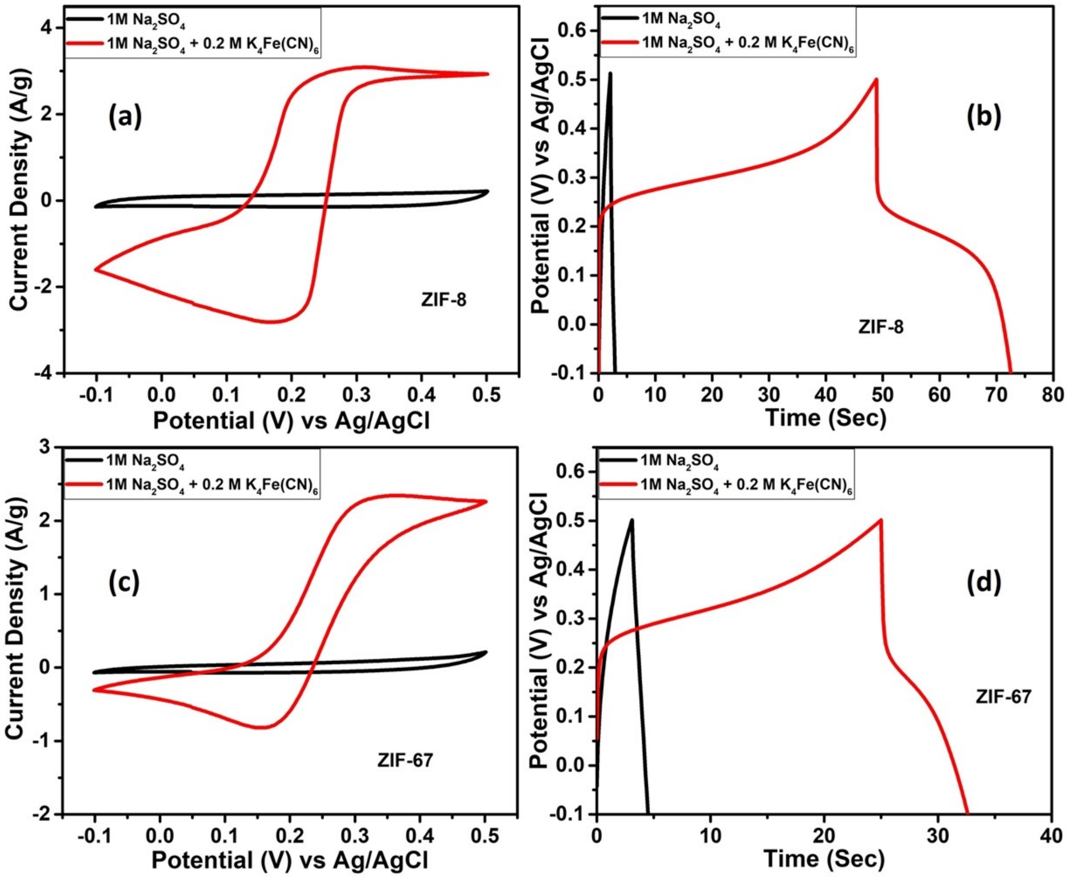

**Figure S3.** (a) CV of ZIF-8 in RAE and 1M $Na_2SO_4$, (b) GCD of ZIF-8 in RAE and 1M $Na_2SO_4$, (c) CV of ZIF-67 in RAE and 1M $Na_2SO_4$, and (d) GCD of ZIF-67 in RAE and 1M $Na_2SO_4$.

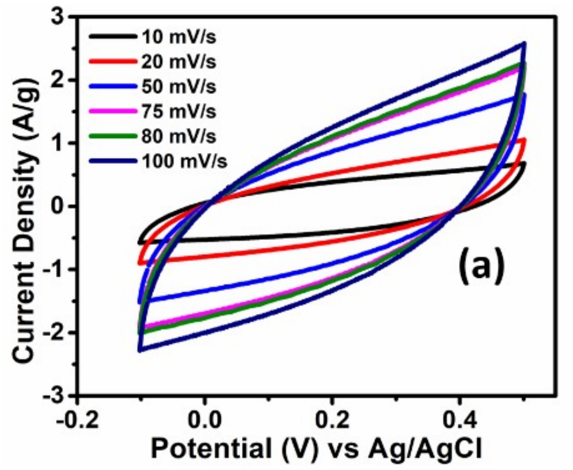


**Figure S4.** (a) CV at different scan rates for Z867 in aqueous electrolyte (1M $Na_2SO_4$).

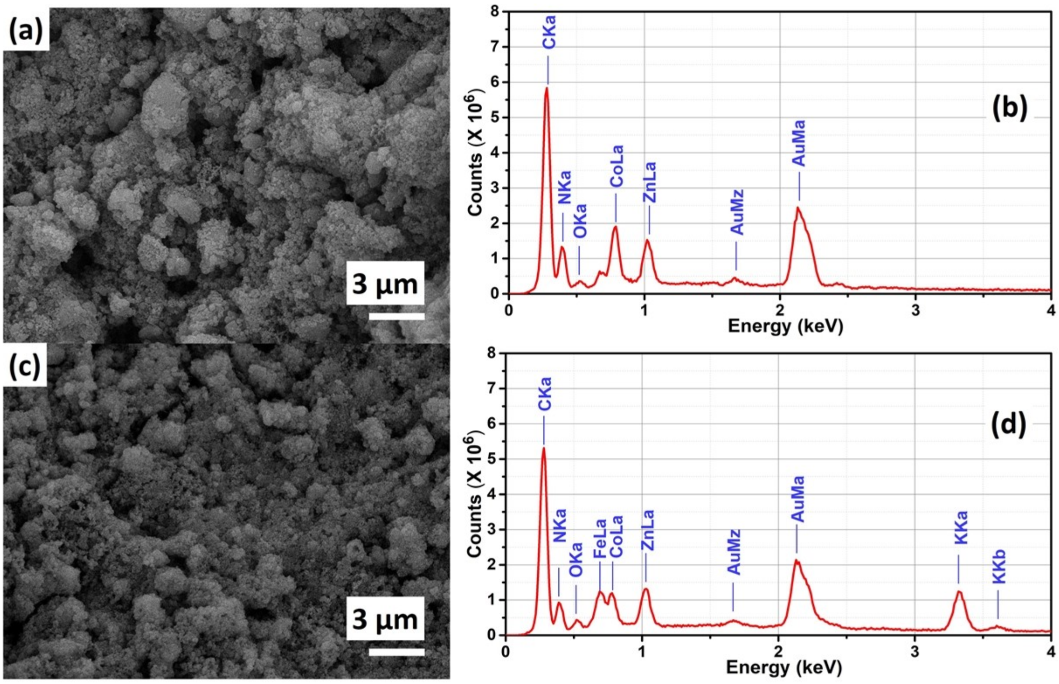

**Figure S5.** Pre and post-cycling characterization of working electrode consists of 80% Z867 material, 10 % PVDF, 10 % carbon black: (a) SEM image before cycling, (b) EDS before cycling, (c) SEM image after cycling, and (d) EDS after cycling using RAE.

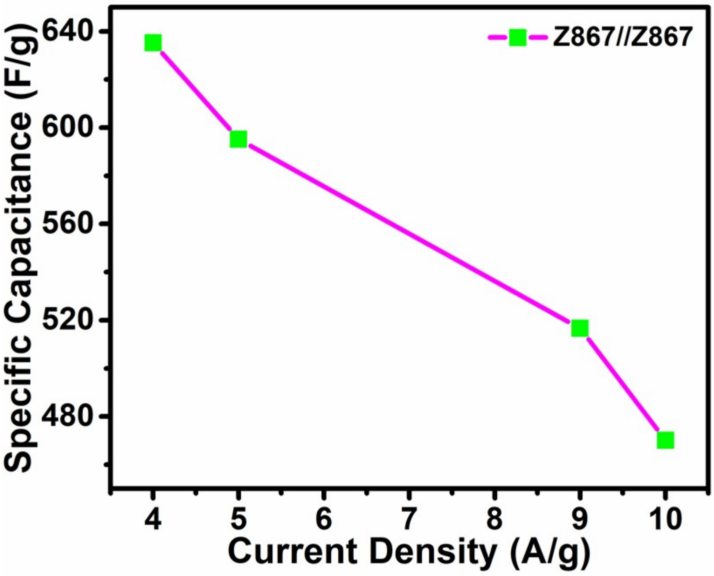


**Figure S6.** Variation of Specific Capacitance with current density for Z867//Z867 redox symmetrical supercapacitor device.

**References-**

(1) Kaur, A.; Shrivastav, V.; Dubey, P.; Deep, A.; Mudahar, I.; Sundriyal, S.; Mishra, S. Waste Paper-Derived Porous Carbon Incorporated with Mesoporous ZIF-8 Crystals for Symmetrical Supercapacitors. *Energy Fuels* **2023**, *37* (15), 11376–11387. https://doi.org/10.1021/acs.energyfuels.3c01528.

(2) Shrivastav, V.; Sundriyal, S.; Goel, P.; Shrivastav, V.; Tiwari, U. K.; Deep, A. ZIF-67 Derived Co3S4 Hollow Microspheres and $WS_2$ Nanorods as a Hybrid Electrode Material for Flexible 2V Solid-State Supercapacitor. *Electrochimica Acta* **2020**, *345*, 136194. https://doi.org/10.1016/j.electacta.2020.136194.

(3) Sundriyal, S.; Shrivastav, V.; Kaur, H.; Mishra, S.; Deep, A. High-Performance Symmetrical Supercapacitor with a Combination of a ZIF-67/rGO Composite Electrode

and a Redox Additive Electrolyte. *ACS Omega* **2018**, *3* (12), 17348–17358. https://doi.org/10.1021/acsomega.8b02065.
